# Development of a LAMP-Based Molecular Species Diagnosis Method for Four Major Agricultural Pests in the Genus *Spodoptera* (Lepidoptera: Noctuidae)

**DOI:** 10.3390/insects12100883

**Published:** 2021-09-29

**Authors:** Hwa Yeun Nam, Ju Hyeon Kim, Si Hyeock Lee, David G. Heckel, Juil Kim

**Affiliations:** 1 Agriculture and Life Science Research Institute, Kangwon National University, Chuncheon 24341, Korea; jessienam@kangwon.ac.kr; 2 Research Institute of Agriculture and Life Science, Seoul National University, Seoul 08826, Korea; biomyst5@snu.ac.kr (J.H.K.); shlee22@snu.ac.kr (S.H.L.); 3 Department of Agricultural Biotechnology, Seoul National University, Seoul 08826, Korea; 4 Department of Entomology, Max Planck Institute for Chemical Ecology, 07745 Jena, Germany; heckel@ice.mpg.de; 5 Program of Applied Biology, Division of Bio-Resource Sciences, College of Agriculture and Life Science, Kangwon National University, Chuncheon 24341, Korea

**Keywords:** *Spodoptera*, mitochondrial genome, invasive pest, LAMP, diagnostic PCR

## Abstract

**Simple Summary:**

Four major *Spodoptera* pests, *S. exigua*, *S. frugiperda*, *S. litura*, and *S. littoralis*, are widely distributed polyphagous pests affecting various crops. Despite different distribution areas, these four species cause serious damage to agriculture worldwide. As these species are morphologically similar at the larval stage, diagnostic methods have been developed and utilized for their identification. Here, we developed a loop-mediated isothermal amplification (LAMP) assay for rapid and effective species diagnosis, along with PCR, to identify Korean field-collected or overseas samples. The optimal conditions for the LAMP assay were 61 °C for 60 min with four LAMP primers. Additional loop primers increased the amplification efficiency in *S. exigua*, whereas increased non-specific amplification was found in other species. A broad range of DNA concentrations was observed in the LAMP assay, and the minimum detectable DNA concentration was 1 pg. The DNA release method for LAMP involved incubation of larval or adult tissue samples for 5 min at 95 °C, without a DNA extraction step. Considering the gradual diversification invasive pest incidence, this simple and accurate LAMP assay can be used for intensive field monitoring of invasive pests and integrated management of these species.

**Abstract:**

Molecular-based species identification tools are helpful to identify tiny insect and lepidopteran pests that show morphological similarities in the larval stage and are essential for quarantine as well as agricultural research. Here, we focused on four major *Spodoptera* pests: *S. exigua*, *S. frugiperda*, *S. litura*, and *S. littoralis*. *S. exigua* and *S. litura* mitochondrial genome sequences were newly identified and species-specific sequence regions were identified in the cytochrome c oxidase subunit II and III regions. Species primers were designed and applied in loop-mediated isothermal amplification (LAMP) and PCR to identify Korean field-collected or overseas samples. The optimal incubation conditions for LAMP were 61 °C for 60 min with four LAMP primers. Additional loop primers increased the amplification efficiency for *S. exigua*, and the nonspecific amplification for other species. The LAMP assay could detect a wide range of DNA concentrations, with the range 1 ng–1 pg in dependence of four LAMP primers. The DNA-releasing technique, without DNA extraction, in the LAMP assay involved larval or adult tissue sample incubation at 95 °C for 5 min. The entire process takes approximately 70 min. This new molecular diagnostic method is simple and accurate, with application in the field and laboratory and for monitoring and ecological studies.

## 1. Introduction

The noctuid genus *Spodoptera* currently includes various agricultural pests; in particular, *S. exigua*, *S. frugiperda*, *S. litura*, and *S. littoralis* are some of the most widely known and invasive pests worldwide. Although the distribution area of each species differs, these four species indisputably cause serious damage to agriculture worldwide. *S. exigua* originated in Southeast Asia and has emerged as an important pest of numerous commercial crops, including cotton, tomato, lettuce, cabbage, and ornamentals [1]. The sporadic outbreaks of *S. exigua* in Korea and evolved resistance to insecticides have been considered as major factors in its spread [2]. An outbreak of *S. frugiperda* was first verified in Africa in 2016. Since then, the range of this outbreak has expanded to Asia and caused serious damage because of its strong adaptability and flight ability [3]. *S. litura* is a prevalent pest in tropical and sub-tropical countries, such as China, Japan, India, Pakistan, and Nepal, causing significant economic losses in vegetables and field crops, including tomato, cauliflower, cabbage, eggplant, cotton, tobacco, and maize [4]. Most insecticides have been widely used to control *S. litura*, but are currently failing to provide adequate control due to the development of insecticide resistance [5]. *S. littoralis* is one of the most notorious and destructive pests of various crops and vegetables and is present in approximately 80 countries across Europe, Asia, and Africa [6]. Although insect control with insecticides is a common strategy, massive insecticide use has caused many populations to acquire strong resistance to most insecticide groups [7].

Even specialists may not be able to accurately diagnose when these pests first occur because of high morphological similarities and substantial color variation at the larval stage. The size of *S. exigua* is slightly smaller compared to the other species. However, the sizes of both larvae and adults of *S. frugiperda*, *S. litura*, and *S. littoralis* are similar, which hinders the identification of these species in the field. Therefore, to solve the limitation of species diagnosis based on morphological characteristics, we attempted to use molecular-based species diagnosis. Most existing molecular species diagnostic methods involve comparison of mtCO1 sequences or PCR [8,9]. Sequencing or PCR-based diagnosis or species diagnosis methods require the samples to be analyzed in a laboratory, which is inconvenient and difficult. In contrast, loop-mediated isothermal amplification (LAMP) has been verified as a method that can be used in the field. Recently, a species diagnosis method was developed for *S. frugiperda* using LAMP [10]. The developed species diagnosis method had high utility in the field, and there was additional demand for the development of a species diagnosis method for related species that occur in the cornfields simultaneously (personal communication) [11]. The four Spodoptera genera used in this study are polyphagous or oligophagous and can damage various crops. There are many cases of their simultaneous occurrence in specific crops, such as in corn fields. For example, *S. exigua*, *S. frugiperda*, and *S. litura* occur simultaneously in corn fields in Korea. Although the occurrence of *S. littoralis* has not yet been confirmed in Korea, its incidence is gradually increasing in Europe. Therefore, in this study, we aimed to develop a diagnostic method for these four major *Spodoptera* species.

## 2. Materials and Methods

### 2.1. Sample Collection and Mitochondrial Genome Sequencing

The larvae of *Spodoptera exigua* lab strains (diamide resistant and susceptible) were reared in the insect pest management lab of Kangwon National University, and were originally from Jindo and National Institute of Agricultural Sciences, Rural Development Administration (RDA), respectively. For mitochondrial genome analysis, a diamide-resistant and -susceptible strain F1 hybrid was used. Additionally, a total of 11 Korean populations were collected as described previously [2]. Field population samples were used to confirm the selected primer sets and LAMP application. The two African populations of *S. frugiperda* originating from Nigeria and Benin, as well as three Korean field populations, were used [10]. The *S. litura* population was collected from a potato field (Gangneung, Korea; 37°46′23″ N, 128°56′08″ E) and used for mitochondrial genome sequencing. For additional Noctuidae pest sample collection, including *S. litura*, *Helicoverpa armigera*, *Agrotis segetum*, and *Mythimna loreyi*, pheromone traps were used with species-specific lures (Green Agrotech, Gyeongsan, Korea), as previously reported [11]. Traps were set in Pyeongchang (37°40′53″ N, 128°43’49″ E), Hongchen (37°43′35″ N, 128°24′33″ E), and Gangneung (37°36′56″ N, 128°45’59″ E) near the corn fields in 2020 and 2021. Most of these species caused frequent or occasional damage in Korean corn fields [12]. *S. littoralis* does not exist in Korea. Therefore, DNA samples were obtained from the Department of Entomology at the Max Planck Institute for Chemical Ecology, Jena, Germany.

Some larvae of the field populations of three species, *S. exigua*, *S. frugiperda,* and *S. litura* were reared in the laboratory for morphological confirmation in the adult stage, and some of individual larvae were directly used for genomic DNA extraction using DNAzol (Molecular Research Center, Cincinnati, OH, USA), followed by quantification with Nanodrop (NanoDrop Technologies, Wilmington, DE, USA). Species were identified by sequencing using universal primers LCO1490 and HCO2198 [8]. For mitochondrial genome sequencing of *S. litura*, the Hiseq platform was used, and more than 2 Gb were sequenced (PHYZEN, Seoul, Korea). The CLC Assembly Cell package (version 4.2.1) was used to assemble the data. After trimming the raw data using the CLC quality trim (version 4.21), assembly was accomplished using the CLC *de novo* assembler with dnaLCW. The assembled sequences were confirmed using BLASTZ [13]. The GeSeq program was used for annotation [14], and the result was manually checked based on alignment with the mitochondrial genomes of other Noctuidae species using MEGA X [15]. In total, five *S. frugiperda* and *S. littoralis* mitochondrial genomes were sequenced and annotated [2]. The mitochondrial genome of *S. exigua* was assembled by whole genome sequencing (Sequence Read Archive: SRP273206, *S. exigua* strain: BAW_Kor-Di-RS1 genome sequencing and assembly) and the subsequent analyses were accomplished in this study.

### 2.2. Phylogenetic Analysis and Primer Design

Molecular phylogenetic analysis of mitochondrial genomes was performed using the maximum likelihood method with bootstrapping, conducted in MEGA X [15,16]. The mitochondrial genome sequences of other Noctuidae species were used in the house DB and downloaded from GenBank, NCBI. For comparative analysis, mitochondrial genomes were aligned using mVISTA [17,18]. Based on the global alignment results, partial sequences were re-aligned for LAMP primer design using PrimerExplorer V5 online software, with some modifications.

### 2.3. LAMP and PCR

The WarmStart^®^ LAMP Kit (New England Biolabs, Ipswich, MA, USA) was used for the LAMP assay. The general protocol of LAMP was carried out according to the manufacturer’s guidelines in a 25 μL reaction. The LAMP conditions were used according to a previous study on *S. frugiperda* [10]. LAMP amplifications were carried out in an Applied Biosystems ProFlex PCR system (ThermoFisher Scientific, Waltham, MA, USA). To optimize the reaction temperature, the LAMP assay was performed at 61, 63, and 65 °C for 60 min with four primers (F3, B3, FIP, and BIP). The efficiency of loop primer(s) was checked in the presence of additional loop primer(s) at 61 °C for 60 min. The detection limit of gDNA was also tested at 61 °C for 60 min using four primers. The DNA release technique was applied under the same conditions as LAMP (61 °C for 60 min with four primers).

For the general PCR, TOYOBO KOD-FX Taq^TM^ (Toyobo Life Science, Osaka, Japan) was used to confirm the species by sequencing and primer checking. Appropriate primers were used with the PCR amplification protocol involving 2 min denaturation at 94 °C followed by 35 cycles of denaturation at 94 °C for 20 s, annealing at 60 °C for 20 s, and extension at 68 °C for 30 s. The amplified DNA fragments were separated using 1.5% agarose gel electrophoresis and visualized with SYBR Green (Life Technologies, Grand Island, NY, USA). Three biological DNA samples were used for each LAMP assay and PCR.

## 3. Results

### 3.1. Mitochondrial Genome Sequencing, Primer Design, and Selection

In total, 15,378 bp of the mitochondrial genome of *S. litura* was verified after trimming from about 2.3 Gb nucleotide sequence information obtained through *Hiseq* (MZ603870). In addition, 15,361 bp of the mitochondrial genome of *S. exigua* was assembled (MT702982). Commonly, two mitochondrial genomes include 13 protein-coding genes: NADH dehydrogenase components (complex I, ND), cytochrome oxidase subunits (complex VI, COX), cytochrome oxidase b (CYPB), and two ATP synthases, two ribosomal RNA genes, and 22 transfer RNAs (Supplementary Figure S1).

Within the mitochondrial (mt) genome results, we assembled the information of various mt genomes registered in GenBank and compared the homology of the nucleotide sequences. MegaBLAST of the mt genomes of four Spodoptera species, including MZ603870 and BAW MT702982, indicated that some intra-species SNPs were identified even in the same species. Based on the phylogenetic analysis, *S. frugiperda* showed the highest intra-species variation among the analyzed Noctuidae pests (Supplementary Figure S2).

**Figure 1:**
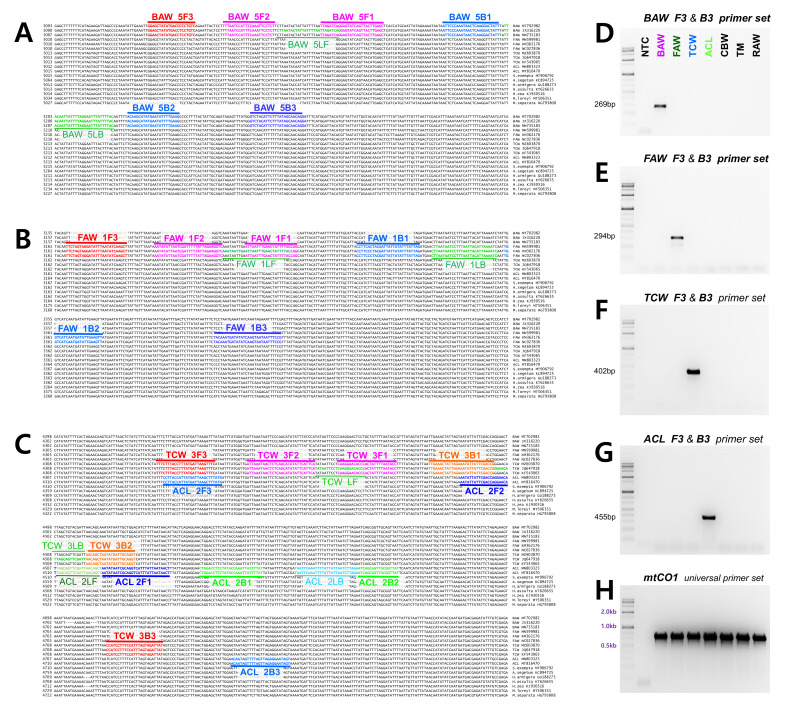
Location of primers and primer binding regions of (**A**) BAW, (**B**) FAW, and (**C**) TCW, ACL on the partial sequences of mitochondrial genomes from five *Spodoptera* species and additional six Noctuidae species. In total, 18 sequence were aligned to pick species-specific regions for primer design. F3 and B3 are the main diagnostic primers. The inner primer, FIP, consists of F1c (complementary sequences of F1) and F2. The other inner primer, BIP is also composed of B1 and B2c (complementary sequences of B2). The four essential LAMP primers (F3, FIP, BIP, and B3) generate the dumbbell structure, and the two loop primers, LF and LB, accelerate the LAMP reaction. Diagnostic external LAMP primers, F3 and B3 were confirmed via PCR (**D**) BAW, (**E**) FAW, (**F**) TCW, (**G**) ACL, and (**H**) PCR with universal primer set (mtCO1) within seven species (BAW, FAW, TCW, ACL, CBW, TM, RAW). NTC: no template control, BAW: Beet armyworm, *S. exigua*, FAW: Fall armyworm, *S. frugiperda*, TCW: Tobacco cutworm, *S. litura*, ACL: African cotton leafworm *S. littoralis*, CBW: Cotton bollworm, *Helicoverpa armigera*, TM: Turnip moth, *Agrotis segetum*, RAW: Rice armyworm, *Mythimna loreyi.*

Thus, we required a primer, which was not mutated within the species and could distinguish between species. Therefore, we designed five sets of LAMP primers for BAW, TCW, and ACL and two sets for FAW, which include a species-specific region that can be used to diagnose the species. Among these primers, the most effective primer set was selected (Table 1). For the selection criteria, 100 ng gDNA of seven species (*S. exigua*, *S. frugiperda*, *S. litura*, *S. littoralis*, *Helicoverpa armigera*, *Agrotis segetum*, and *Mythimna loreyi*), for which the species was confirmed using the mtCO1 universal primer set, was added and incubated at 61 °C for an hour. Among three different incubation temperature conditions (65, 63, and 61 °C), the best results were obtained at 61 °C in most of the targeted four Spodoptera species diagnostic primer sets (data not shown). Then, we selected the primers that definitively diagnosed the species. Finally, we selected primers that clearly revealed the results of species diagnosis by general PCR (data not shown). Through this selection, set 5 was selected for BAW (cytochrome c oxidase subunit III part), set 1 for FAW (cytochrome c oxidase subunit II part), set 3 for TCW, and set 2 for ACL. All species diagnosis primers were selected in the region encoding ATP synthase F0 subunit part 6 and cytochrome c oxidase subunit III (Figure 1). The selected primers can be utilized for LAMP as well as for general PCR (Figure 1). Amplification of mtCO1 markers, used as a positive control, performed well in all species of various samples used in the experiment.

### 3.2. Comparison of Loop Primer Effectiveness

**Figure 2:**
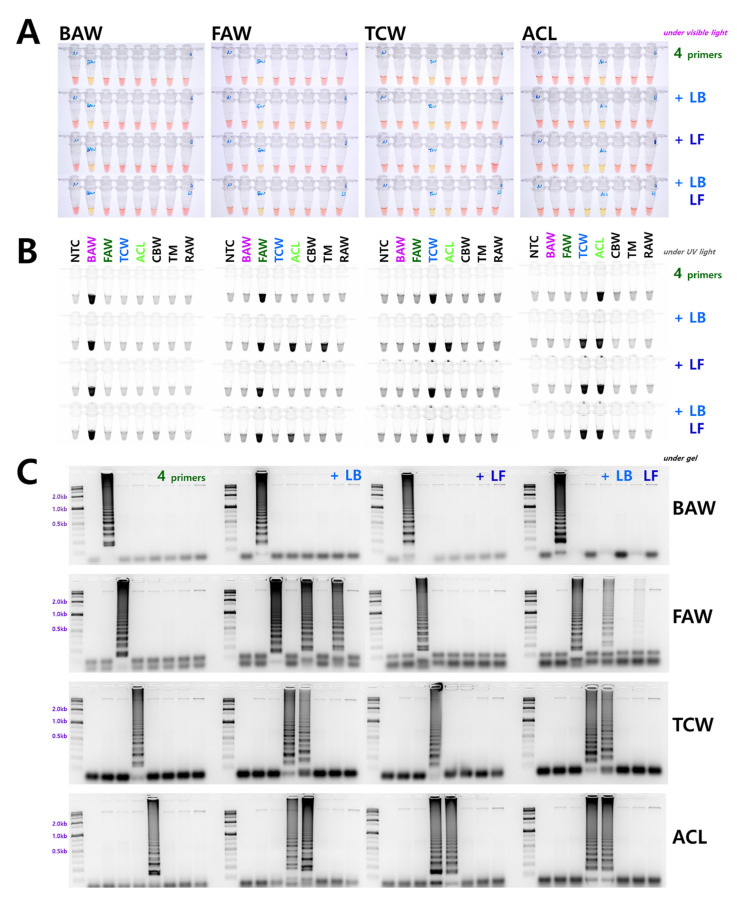
LAMP assay results with four primers (F3, B3, FIP, and BIP) and additional loop primers, loop backward (LB) and loop forward (LF). The LAMP assay was performed at an incubation temperature of 61 °C for 60 min under (**A**) visible light, (**B**) ultraviolet light with SYBR, and (**C**) gel electrophoresis. NTC: no template control, BAW: Beet armyworm, *S. exigua*, FAW: Fall armyworm, *S. frugiperda*, TCW: Tobacco cutworm, *S. litura*, ACL: African cotton leafworm *S. littoralis*, CBW: Cotton bollworm, *Helicoverpa armigera*, TM: Turnip moth, *Agrotis segetum*, RAW: Rice armyworm, *Mythimna loreyi*.

In addition to the four essential primers selected (F3, B3, FIP, and BIP), we verified whether the use of additional loop primers could increase the reaction efficiency by adding each loop primer individually (LB or LF) and both primers simultaneously (LB and LF). This resulted in non-specific reactions except in the case of BAW, in which primer addition increased the reaction efficiency. In particular, the reaction efficiency was significantly improved when the two loop primers were added and reacted simultaneously. A representative electrophoresis image is shown in Figure 2C. Contrary to BAW, non-specific reactions occurred with loop primer addition in FAW, TCW, and ACL, and most of these occurred in TCW and ACL, which have high nucleotide sequence homology (Figure 2). A non-specific reaction occurred in a species belonging to another genus with low sequence homology. When LB was added to FAW, the reaction indicated another genus, *Agrotis segetum*. We examined more than five replicates (biological replicates using gDNA samples from various local populations) for this result, and gel electrophoresis results were similar except for slight differences in intensity.

### 3.3. Determining the Diagnosis Limit Concentrations and Combining the DNA Release Technology

**Figure 3:**
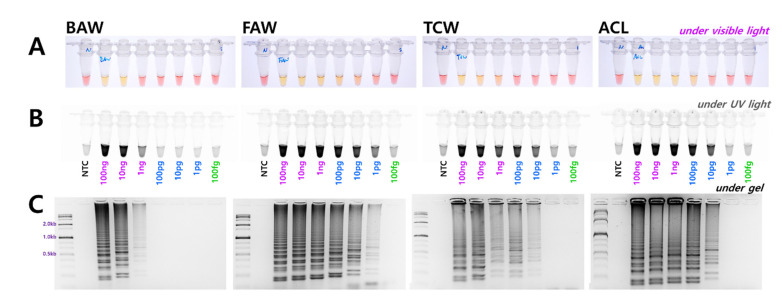
Identification of the genomic DNA detection limit in the four *Spodoptera* species diagnostic LAMP assay from 100 ng to 100 fg using four LAMP primers under (**A**) visible light, (**B**) ultraviolet light with SYBR Green, and (**C**) gel electrophoresis. NTC: no template control.

**Figure 4:**
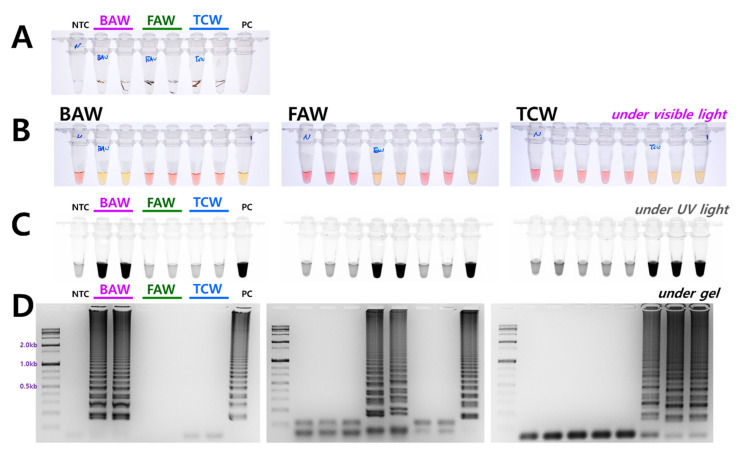
Representative LAMP assay results with the DNA release technique from (**A**) insect tissue. Around 10 mg of larval tissue or adult leg (or antenna) was incubated at 95 °C for 5 min. LAMP assay results with four primers (F3, B3, FIP, and BIP) (**B**) under visible light, (**C**) ultraviolet light with SYBR Green, and (**D**) gel electrophoresis. The tubes in panel B and C and vertical lines in panel D have the same order of the species of DNA. NTC: no template control, PC: positive control.

The diagnostic concentrations were sequentially decreased by 1/10 from 100 ng to 100 fg in a 25 µL standard reaction solution for four species. The results indicated that BAW was detected up to 1 ng, FAW up to 1 pg, and TCW and ACL were detected up to 10 pg concentrations (Figure 3). Although the diagnostic limit concentration was different in the 1 h equivalent, the reaction was conducted. These standards are based on the electrophoresis results; the diagnostic limit concentration could, thus, be somewhat higher by visual observation or upon confirmation after UV treatment. The reaction proceeds smoothly at various concentrations despite differences between species. Therefore, a method was developed without a separate DNA extraction process that could be combined with the LAMP method. A part of the antenna or leg tissue from BAW, FAW, and TCW adults or larvae was cut and reacted at 95 °C for 5 min to release DNA, similar to the method reported previously for FAW [10]. By measuring the amount of released DNA using a nanodrop system, the DNA concentration was found to be at least 1 ng or more, even though the concentration differed depending on the amount of tissue in the initial reaction. Overall, sufficient DNA was released for the reaction, and showed the same results as the positive control (DNA extracted using DNAzol) for BAW, FAW, and TCW (Figure 4).

## 4. Discussion

Species diagnosis using molecular biology tools is often based on changes in small nucleotide sequences, thus necessitating appropriate primer design. Based on the mt genome information in GenBank for species collected from various countries and the mt genome information analyzed by ourselves, we focused on the part without any mutations within the species that can be distinguished from other species, and designed a diagnostic primer. As the diagnostic method for FAW has already been developed, additional primer design was extremely limited. Therefore, two additional primer sets were designed for FAW, and five different parts targeting primers were designed for BAW, TCW, and ACL. The set with the highest diagnostic efficiency was selected (Figure 1). The most basic selection criterion was that when the reaction was performed using four essential primers, all three repetitions were diagnosed in a species-specific manner, and the results could be confirmed within 1 h.

A substantial problem in designing primers within the mt genome is its AT-rich nature. However, the length of the primer or the LAMP product may be increased to adjust the primer melting temperature (Tm). Among the LAMP primers designed in this study, based on the F3 and B3 primer sets, set 5 of the TCW LAMP primer had a length of 919 bp, and its reaction time was longer than an hour. The ACL set 5 was not selected because its product size was 856 bp, and the reaction time was more than 1 h (approximately 1.5 h). Thus, a common characteristic of the selected primer sets was that the product size was within 500 bp. Furthermore, at least two species-specific nucleotides were adjacent to the 3′ end of the primer, except for TCW, and in TCW, only one species-specific nucleotide was present at the 3′ end of the F3 and B3 primers. Nevertheless, the presence of two or more species-specific nucleotides showed more effective diagnostic results compared to other primer sets adjacent to the 3′ end of the primer.

In the case of allele-specific LAMP (AS-LAMP), which was attempted in some studies, the primer was designed to enable trait-specific diagnosis within the inner primer [19,20]. Therefore, we consider that AS-LAMP diagnosis is possible based on F3 and B3 primers, depending on the conditions. Adding a loop primer was reported to shorten the reaction time and increase the reaction efficiency compared to the addition of four essential primers [21]. With the loop primer alone, the reaction efficiency could be increased if the species specificity was high. Otherwise, we considered that a non-specific reaction occurred when the nucleotide sequence was present in many species.

TCW and ACL are representative cases in which the mt genome homology is very high, ranging from 97.99% to 98.05%, and a reaction occurred in both species with the addition LB (Figure 2). BAW, despite belonging to the *Spodoptera* genus, shows low homology in the mt genome (standard of BAW, ACL 94.71%, TCW 94.21%, FAW 93.73%), and its reaction efficiency increased when a loop primer was added to the reaction. The increase in the reaction efficiency was particularly evident in the electrophoresis results (Figure 2C). The use of loop primers in diagnosing closely related species using LAMP does not unconditionally increase the reaction efficiency. Therefore, accurate verification is required to determine whether a non-specific reaction may occur in a related species. Based on the equivalent time standard, the diagnostic limit concentration differed depending on the primer set of each species. This depends on the LAMP reaction efficiency regardless of the length of the reaction product, based on the F3 and B3 primers (Figure 1 and Figure 2). As the LAMP reaction was performed stably at various concentrations, we applied the DNA release method to LAMP, which replaced the existing DNA extraction, by reacting the samples at 95 °C for 5 min in 20 μL of nucleotide water. The DNA release method decreased the reaction time compared to the general process using gDNA obtained by DNA extraction. When LAMP can be processed without a separate DNA extraction process, when a thermoregulator (heat block, etc.) is available, a tissue sample from the insect is placed in a tube to be diagnosed in the field, and reacted at 95 °C for 5 min. Then, a part of the resulting supernatant (2 μL) is added to the LAMP reaction solution (master mix) together with the primer and reacted at 61 °C for one hour to confirm the result (Figure 4). This method can be used for simple species identification as well as for comprehensive ecological understanding, such as species distribution and occurrence, and for establishing a systematic construction of integrated pest management for pests from the genus *Spodoptera*.

## 5. Conclusions

In this study, a species diagnosis marker was designed within the mt genome and combined it with a DNA release method that is highly applicable for species diagnosis in the field. Moreover, the entire process takes approximately 70 min. In addition to the four essential primers selected (F3, B3, FIP, and BIP), only in the case of *S. exigua*, adding loop primers (LB and LF) significantly increased the reaction efficiency. This simple and accurate diagnosis method using the LAMP assay could be applied in intensive field monitoring and management of four *Spodoptera* pest species: *S. exigua, S. frugiperda*, *S. litura*, and *S. littoralis*. In addition to the four essential primers selected (F3, B3, FIP, and BIP), only in the case of *S. exigua*, primer addition increased the reaction efficiency.

## Figures and Tables

**Table 1:** Primers used for species diagnostic LAMP and PCR in this study.

Purpose	Primers	Sequence (5’ → 3’)
*for Spodoptera exigua (beet armyworm, BAW)*		
	BAW_5F3	GGAGCTATATGACCCCCT**GTC **^1^
	BAW_5B3	TCCTGTTGCTATAAAGAATGTAGA**C**
	BAW_5FIP	GCTCAAGTAACTGATACTCCTGATCTAATTTAATCCATTTCAAATTCCTCT
	BAW_5BIP	ATTCCCAAATAACTCAAGGACTATTCTTCAAAATATTCATATGCTTGT
	BAW_5LF	CTGATCTAATTAAAATAATAGTATTAAG
	BAW_5LB	TTACAATTATTTTAGGAATTTATTTTAC
*for Spodoptera frugiperda (fall armyworm, FAW)*		
	FAW_1F3	TTCTAGTAGGATATTTAATATCAAG**CT**
	FAW_1B3	CGAAAATTATTACTTGATATATCATTTG**TA**
	FAW_1FIP	CTGGTAAAATAGTTCAAATTAATAATATATTAATCGATTTTTATTAGAAGGTC
	FAW_1BIP	CCTTCACTACGATTATTATATTTATTAGAACTTCAATATCATTGATGACCA
	FAW_1LF	GTAAAATAGTTCAAATTAATTCAATTATTT
	FAW_1LB	CTTAATAATCCTTTAATTACATTAAAATC
*for Spodoptera litura (tobacco cutworm, TCW)*		
	TCW_3F3	GTTTCTTTACCTTTATGATTAAGTTT**C **^2^
	TCW_3B3	ATAATCTACTAAATGGAAAGGATG**G**
	TCW_3FIP	GGTATTAAAATAGTGGGTGTTCCTTGATTAAATAACTCTCAACATATATTCATTCA
	TCW_3BIP	TTGAAACTATTAGAAATATTATTCGACCGACCTGCAATCATATTAGCTGTT
	TCW_3LF	GGGTGTTCCTTGAGGAATTATA
	TCW_3LB	GGGAACATTAGCAGTTCGATT
*for Spodoptera littoralis (African cotton leafworm, ACL)*		
	ACL_2F3	CCTTACCATTATGATTAAG**C**TT**T**AT**G**
	ACL_2B3	GACTATTCCTCTAACTAAAACTATT**GT**
	ACL_2FIP	AGTTATTAATAAATGACCTGCGATTATATTAATATTATTCGACCAGGAACAT
	ACL_2BIP	CTGGACCTTCTATACCAAATTATTTTATATAACCGCAACTGCTGAT
	ACL_2LF	AGCTGTTAATCGAACTGCTA
	ACL_2LB	TAATTCAAATTTTATTATTAATCTTAGA
*for PCR*		
	LCO1490	GGTCAACAAATCATAAAGATATTGG
	HCO2198	TAAACTTCAGGCTGACCAAAAAATCA

^1^ Red and bold letters indicate species specific sequences for the diagnostic reactions. ^2^ ‘G’s at the 5’ end depicted in pink were added to adjust the primer melting temperature.

## Supplementary Materials

The following are available online at https://www.mdpi.com/article/10.3390/insects12100883/s1, Figure S1: Organization of the mitochondrial genome of the Korean population of *Spodoptera exigua* (MT702982) and *S. litura* (MZ603870). ND: NADH dehydrogenase components (complex I) in yellow. COX: Cytochrome oxidase subunits (complex VI) in pink. ATP synthase in green. CYPB: cytochrome oxidase b in purple. Ribosomal RNA genes are shown in red and tRNA genes are shown in blue. Noncoding regions are not colored; Figure S2: Phylogenetic relationship of some Noctuidae genera inferred using the neighbor-joining method based on the whole mitochondrial genome sequence in MEGA-X. The percentage of replicate trees in which the associated taxa clustered together in the bootstrap test (1000 replicates) is shown next to the branches. Evolutionary distances were computed using the maximum composite likelihood method and are indicated in units of the number of base substitutions per site. This analysis involved 31 nucleotide sequences. *Pieris rapae* (Lepidoptera: Pieridae) was used as the experimental group.
