# Supplementary material for: Development of a LAMP-Based Molecular Species Diagnosis Method for Four Major Agricultural Pests in the Genus Spodoptera (Lepidoptera: Noctuidae)
Source: Insects. 2021 Sep 29;12(10):883. doi: 10.3390/insects12100883 (PMC8541213; doi:10.3390/insects12100883)
Supplement: Supplementary file 1 [file insects-12-00883-s001.zip › insects-1382267-supplementary.pdf]

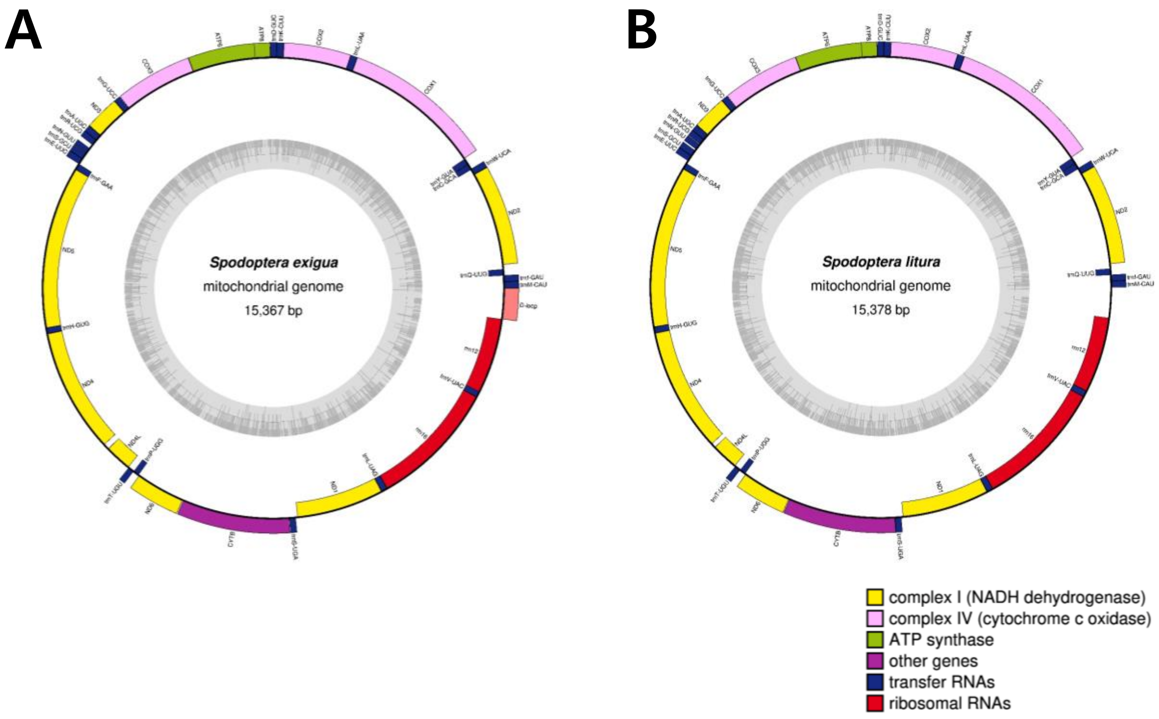

Figure S1. Organization of the mitochondrial genome of Korean population of *Spodoptera exigua* (MT702982) and *S. litura* (MZ603870). ND: NADH dehydrogenase components (Complex I) in yellow. COX: cytochrome oxidase subunits (Complex VI) in pink. ATP synthase in green. CYPB: cytochrome oxidase b in purple. Ribosomal RNA genes in red, tRNA genes in blue. Noncoding regions are not colored.

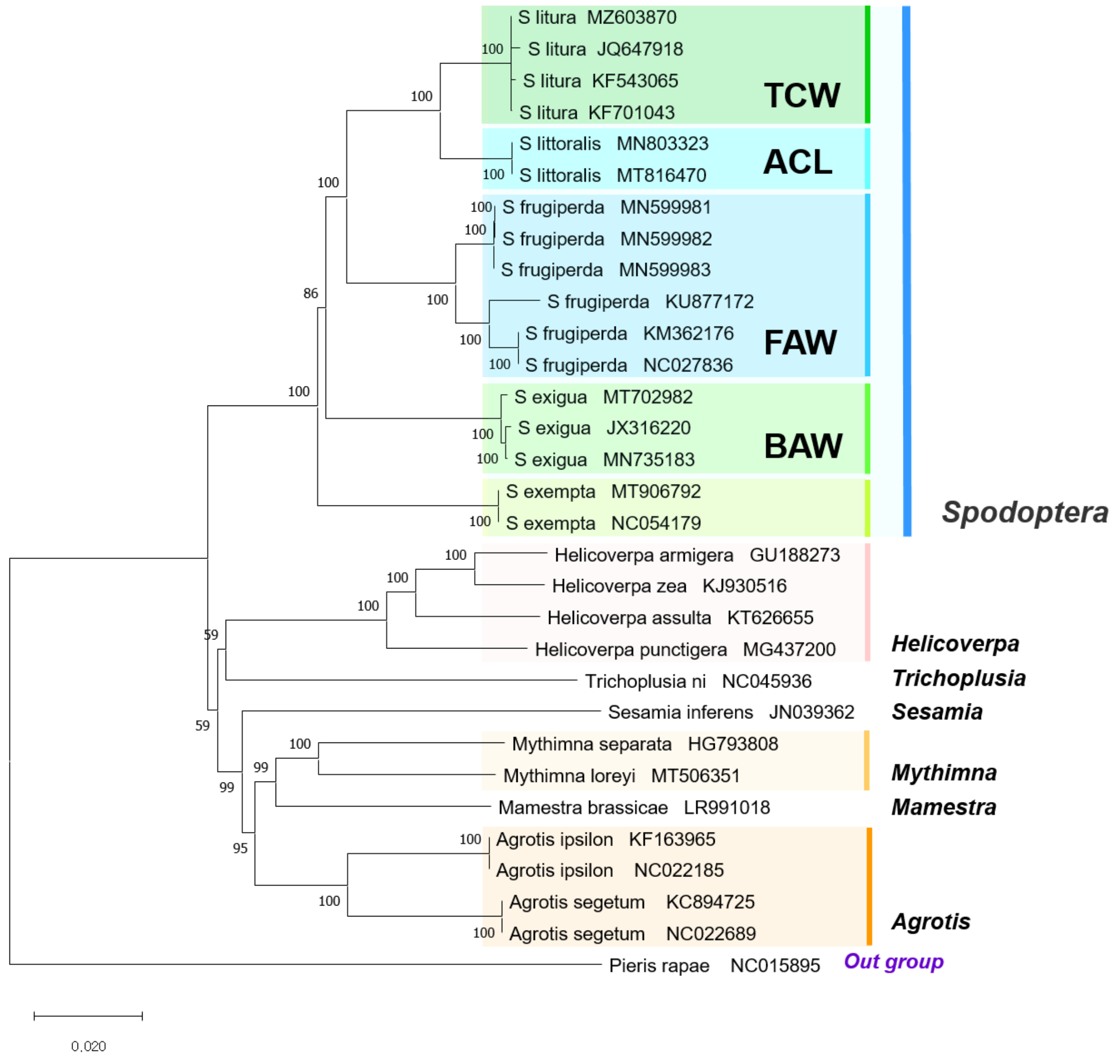

Figure S2. Phylogenetic relationship of some Noctuidae genius inferred using the Neighbor-Joining method based on the whole mitochondrial genome sequence under MEGA-X. The percentage of replicate trees in which the associated taxa clustered together in the bootstrap test (1,000 replicates) are shown next to the branches. The evolutionary distances were computed using the Maximum Composite Likelihood method and are in the units of the number of base substitutions per site. This analysis involved 31 nucleotide sequences. *Pieris rapae* (Lepidoptera: Pieridae) was used out group.
